# Regional differences in triage decisions affect hospital mortality among frail COVID-19 patients in the COvid MEdicaTion study

**DOI:** 10.1186/s12879-025-10540-2

**Published:** 2025-02-04

**Authors:** Julia Minnema, Roos Sablerolles, Janneke van Kempen, Hugo van der Kuy, Harmke Polinder-Bos, Bob van de Loo, Jorie Versmissen, Miriam L. Haaksma, Melvin Lafeber, Miriam C. Faes, on behalf of the COMET research team^, Julia Minnema, Roos Sablerolles, Janneke van Kempen, Hugo van der Kuy, Harmke Polinder-Bos, Bob van de Loo, Jorie Versmissen, Melvin Lafeber, Miriam C. Faes, Ingrid van Haelst, Louise Andrews, Marleen Kemper, Roland van den Berg, Elise Slob, Firdaouss Boutkourt, Erik van Kan, Ronald Van Etten, Mariette Kappers, Peter van Wijngaarden, Anja Vos, Linda Hendriksen, Hugo de Wit, Loes Visser, Judith Derijks, Doris Haider, Nikolaus Lindner, Monika Schwap, Jos Tournoy, Lorenz Van der Linden, Morten Baltzer Houlind, Roberto Tessari, Paola Chessa, Marco Gambera, Isabella Martignoni, Laura Agnoletto, Margarida Falcao, Helena Farinha, Dina Mendes, Ines Carmo, Joana Soares, Fatima Falcao, Mariana Solano, Erica Viegas, Marta Miarons, Maria Queralt Gorgas, Cristina García Yubero, Kim Blum, Kim Keijzers, Silke Lim

**Affiliations:** 1 Department of Internal Medicine, Erasmus MC, University Medical Center Rotterdam, Rotterdam, the Netherlands; 2 Department of Hospital Pharmacy, Erasmus MC, University Medical Center Rotterdam, Rotterdam, the Netherlands; 3 Department of Geriatrics, Amphia Hospital, Breda, the Netherlands; 4 Digitalis Rx BV, Amsterdam, the Netherlands; 5 Department of Public Health and Primary Care, Leiden University Medical Centre, Leiden, The Netherlands; 6 University Network for the Care sector South-Holland, Leiden University Medical Center, Leiden, The Netherlands; 7 Noordwest Ziekenhuisgroep, Alkmaar, the Netherlands; 8 Meander MC, Amersfoort, the Netherlands; 9 Amsterdam UMC, AMC, Amsterdam, the Netherlands; 10 Farmadam Apotheek, Amsterdam, the Netherlands; 11 Gelre ziekenhuizen, Apeldoorn/Zutphen, the Netherlands; 12 Treant Zorggroep, Emmen, the Netherlands; 13 Tergooi hospital, Hilversum, the Netherlands; 14 Canisius Wilhelmina Hospital, Nijmegen, the Netherlands; 15 Haga Ziekenhuis, Den Haag, the Netherlands; 16 Radboud UMC, Nijmegen, the Netherlands; 17 Klinik Favoriten, Vienna, Austria; 18 University Hospitals Leuven, Leuven, Belgium; 19 Copenhagen University Hospital Amager and Hvidovre, Copenhagen, Denmark; 20 IRCCS Ospedale Sacro Cuore Don Calabria, Negrar di Valpolicella, Italy; 21 San Francesco Hospital Nuoro, Nuoro, Italy; 22 Pederzoli Hospital, Peschiera del Garda, VR Italy; 23 Azienda Ulss 5 Veneto, Rovigo, Italy; 24 Egas Moniz Hospital, Lisboa, Portugal; 25 Hospital São Francisco Xavier, Lisboa, Portugal; 26 Vall d’Hebron Hospital, Barcelona, Spain; 27 University Hospital Infanta Sofía, San Sebastián de los Reyes, Spain; 28 FHP Kantonsspital Aarau, Aarau, Switzerland; 29 Cantonal Hospital of Lucerne, Lucerne, Switzerland

**Keywords:** COVID-19, Frailty, Triage, Policy

## Abstract

**Objectives:**

The scarcity of intensive care unit (ICU) beds during the COVID-19 pandemic has led to a large number of national and international guidelines for the triage of ICU admission. Regional variation in medical decision making might affect ICU triage decisions. We investigate whether regional differences in ICU admission, as surrogate for triage decisions, affect in-hospital mortality in COVID-19 patients.

**Methods:**

The COMET study is a multicenter, observational cohort study, including adult patients hospitalized for COVID-19 between March 2020– July 2020. Patients’ characteristics, prescribed medication, clinical characteristics, and CFS were collected. Patients from 11 European countries were included and these countries were categorized into two regions: north and south. The effects of region on ICU admission and in-hospital mortality were assessed using logistic regression analyses stratified for frailty.

**Results:**

Frail patients had a higher risk for ICU admission in southern compared to northern countries (OR: 1.64; 95%CI: 1.10–2.46), whereas fit patients had a similar risk for ICU admission in southern compared to northern countries (OR: 0.75; 95%CI: 0.55–1.01). There was no difference in in-hospital mortality between northern and southern countries for fit and frail patients (respectively OR: 0.82; 95% CI 0.52–1.29, and OR: 1.11; 95% CI: 0.74–1.66).

**Conclusion:**

Our study shows that, despite variation in rates of ICU admission between northern and southern countries for frail patients, no difference in in-hospital mortality was observed. This might help optimize prioritization of resources in a pandemic setting while offering options for palliative care instead of ICU admission.

**Supplementary Information:**

The online version contains supplementary material available at 10.1186/s12879-025-10540-2.

## Introduction

During the first wave of the COVID-19 pandemic, scarce hospital resources raised ethical dilemmas about triage decisions, predominantly for admission to the intensive care unit (ICU). In most countries, hospitals were overcrowded and largely unprepared for the high number of COVID-19 referrals and ICU admissions. European countries differ in ICU policies regarding number of ICU beds per head of population but also in medical decision making to determine who to admit to the ICU [1]. The aim of triage is to utilize scarce resources effectively and efficiently. To prioritize ICU admission, guidelines were created to support bedside decision making for the triage of COVID-19 patients [2, 3]. All European triage guidelines share a common principle of maximizing the benefit of available resources, although the operationalization varies per country.

Prior to the COVID-19 pandemic, it was recognized that regional variations exist in end-of-life practices within the ICU, which may be attributed to social, religious and/or legal reasons [4]. Following the COVID-19 pandemic, a multicenter, prospective study, including 3105 critically ill patients aged 70 years and older within the ICU in Europe, observed that there is a gradient in rates of treatment limitation from north to south [5]. In Northern European countries, the highest frequency of treatment limitation was reported (48%), followed by Central European countries (39%), and the lowest rates were seen in Southern European countries (24%). This gradient reflects a decreasing incidence of withholding and withdrawing treatment from north to south. Considerations to decline ICU admission are in general a poor prognosis based on patient characteristics such as age, pre-existing comorbidities, and/or acute organ failure [5]. Throughout the pandemic, explicit consideration of frailty has become more common through the use of the Clinical Frailty Scale (CFS) [6, 7]. In addition, differences in triage guidelines between northern and southern European countries influence decisions regarding ICU admission. For instance, in case of a shortage in ICU beds, policymakers in the Netherlands argue that it is justifiable to, after an assessment based on prognosis and chance of survival, evaluate patient priority for ICU admission based on the expected length of stay on the ICU [2]. Conversely, this factor is not described in other European guidelines as part of any triage rule. In this study, we aim to assess whether regional differences in ICU admission, as surrogate for triage decisions, affect in-hospital mortality in COVID-19 patients.

## Methods

### Study design and participants

The COMET study is an observational, retrospective cohort study conducted during the initial wave of the COVID-19 pandemic (March– July 2020). The study included patients ≥ 18 years who had a high clinical likelihood of COVID-19 based on clinical, biochemical and/ or radiological criteria or tested positive for severe acute respiratory syndrome coronavirus 2 (SARS-CoV-2) and were subsequently admitted to the hospital. The rationale and design of the COMET study have previously been described [8, 9]. In this post-hoc analysis of the COMET study, patients with available CFS score coming from 63 centers in 11 European countries were included. These countries were categorized into two regions: north (Austria, Belgium, Switzerland, Germany, Denmark, Great Britain, the Netherlands, and France) and south (Italy, Portugal and Spain). The differentiation between northern and southern countries in our analysis was based on geographical location and supported by findings from previous studies highlighting significant regional differences in medical decision-making, particularly regarding treatment limitation at the ICU [5, 10]. The members of the COMET research group are listed in Supplemental List 1. The Erasmus Medical Center Medical Ethics Review Committee in Rotterdam, the Netherlands, approved the study (MEC-2020-0277), and each institutional review board of the participating hospitals approved the use of data, as described in the protocol [8].

### Data collection

Data collection included prescribed medication, patient characteristics at admission i.e. the clinical frailty scale (CFS), and clinical outcomes such as ICU admission and in-hospital mortality [8, 9]. To ensure a consistent definition of ICU beds across the participating centers, we verified whether their ICU beds adhered to a standardized definition, which defines ICU beds as ‘beds with critical care equipment (e.g., ventilator) that are adequately staffed by specialized nurses and physicians with critical care training’ [11]. Data were retrospectively collected from the electronic medical records in an online database (Clinical Rules reporter, version 1.6.3; Digitalis Rx, Amsterdam, the Netherlands) and pseudonymized.

### Statistical analysis

The effect of region on ICU admission and in-hospital mortality were assessed using logistic regression analyses stratified for frailty (fit: CFS 1–3, mildly frail: CFS 4–5, frail: CFS 6–9). Two different models were built.

- Model 1: A crude, unadjusted estimate for region.
- Model 2: Model 1 and an additional adjustment for age and sex.

Sensitivity analyses were performed in which we adjusted the classification of region. Since the allocation of central European countries (Austria, Switzerland, Germany and France) is considered arbitrary, we have assigned them to the southern countries in the sensitivity analyses, whereas they are classified as northern countries in the primary analyses [5].

## Results

Data were collected for 2434 patients ≥18 years included in 63 centers in 11 European countries (North: Austria *n* = 447, Belgium *n* = 46, Switzerland *n* = 91, Germany *n* = 12, Denmark *n* = 30, Great Britain *n* = 53, the Netherlands *n* = 1030, France *n* = 71; South: Italy *n* = 327, Portugal *n* = 148, Spain *n* = 179).

Baseline characteristics for patients in northern and southern countries were compared in Table 1. Patients in the northern and southern countries were of similar age (resp. mean 67 [IQR 55–77] vs. mean 68 [IQR 56–78]) and used a similar number of prescribed drugs (resp. mean 3 [IQR 1–7] vs. mean 3 [IQR 1–6).

**Table 1:** Characteristics for patients in northern and southern countries (*n* = 2434)

	Total (*n* = 2434)	North (*n* = 1780)	South (*n* = 654)
**Patient characteristics**			
Age (years), median [IQR]	67 (55–77)	67 (55–77)	68 (56–78)
Men, n (%)	1480 (60.8)	1094 (61.5)	386 (59.0)
BMI, n (%)			
Normal	798 (41.2)	543 (36.5)	255 (56.4)
Pre-obesity	564 (29.1)	469 (31.5)	95 (21.0)
Obesity	577 (29.8)	475 (31.9)	102 (22.6)
Frailty, n (%)			
Fit (CFS 1–3)	1377 (56.6)	1043 (58.6)	334 (51.1)
Mildly frail (CFS 4–5)	564 (23.2)	428 (24.0)	136 (20.8)
Frail (CFS 6–9)	493 (20.3)	309 (17.4)	184 (28.1)
**Use of medication pre-admission**			
Blood pressure-lowering drugs, n (%)	1136 (53.3)	790 (44.4)	346 (52.9)
Antiplatelet drugs, n (%)	405 (16.6)	297 (16.7)	108 (16.5)
Oral anticoagulant drugs, n (%)	272 (11.2)	223 (12.5)	49 (7.5)
Glucose-lowering drugs, n (%)	437 (18.0)	318 (17.9)	119 (18.2)
Antipsychotic drugs and cholinesterase inhibitors, n (%)	143 (5.9)	85 (4.8)	58 (8.9)
Number of prescribed drugs, mean [IQR]	3 (1–7)	3 (1–7)	3 (1–6)
**In-hospital outcomes**			
Intensive care admission, n (%)	616 (25.3)	454 (25.5)	162 (24.8)
In-hospital mortality, n (%)	456 (18.7)	343 (19.3)	113 (17.3)

Legend: North: Austria, Belgium, Switzerland, Germany, Denmark, Great Britain, the Netherlands, France; South: Italy, Portugal, Spain

Non-available values: Total: 495 BMI; North: 293 BMI; South: 202 BMI

The association between region and ICU admission stratified for frailty score is shown in Table 2. After adjustments of covariates (model 2), frail patients had a higher risk for ICU admission in southern compared to northern countries (OR: 1.64 (95% CI: 1.10–2.46)). Fit patients had similar risk for ICU admission in southern compared to northern countries (OR: 0.75 (95% CI: 0.55–1.01)), although this was borderline. For mildly frail patients, also no difference was observed in the risk for ICU admission between northern and southern countries (OR: 0.86 (95% CI: 0.51–1.46)).

**Table 2:** Logistic regression analysis for region and respectively ICU admission and in-hospital mortality

		ICU admission	In-hospital mortality
	North	South OR (95% CI)	South OR (95% CI)
**Fit (CFS 1–3) (** *n* ** = 1377)**			
Model 1: crude analysis	Ref.	0.71 (0.53–0.96)	0.71 (0.46–1.10)
Model 2: model 1 + adjusted for age and sex	Ref.	0.75 (0.55–1.01)	0.82 (0.52–1.29)
**Mildly frail (CFS 4–5) (** *n* ** = 564)**			
Model 1: crude analysis	Ref.	0.82 (0.49–1.36)	0.25 (0.13–0.47)
Model 2: model 1 + adjusted for age and sex	Ref.	0.86 (0.51–1.46)	0.16 (0.08–0.33)
**Frail (CFS 6–9) (** *n* ** = 493)**			
Model 1: crude analysis	Ref.	1.52 (1.03–2.23)	1.25 (0.86–1.82)
Model 2: model 1 + adjusted for age and sex	Ref.	1.64 (1.10–2.46)	1.11 (0.74–1.66)

Abbreviations: OR, odds ratio; CI, confidence interval; North: Austria, Belgium, Switzerland, Germany, Denmark, Great Britain, the Netherlands, France; South: Italy, Portugal, Spain

The association between region and in-hospital mortality stratified for frailty score is shown in Table 2. After adjustment for covariates (model 2), no difference in in-hospital mortality was observed between northern and southern countries for fit and frail patients (respectively OR: 0.82; 95% CI 0.52–1.29, and OR: 1.11; 95% CI: 0.74–1.66). Mildly frail patients had a decreased risk on in-hospital mortality in southern compared to northern countries (OR: 0.16; 95% CI: 0.08–0.33).

The sensitivity analyses for the association between region and ICU admission, and region and in-hospital mortality in shown in Supplemental Table 1. Contrary to the primary analyses, a lower risk was observed for ICU admission and in-hospital mortality in fit patients in northern compared to southern countries (resp. OR: 0.44, 95% CI: 0.34–0.56 and OR: 0.68; 95% CI: 0.47–0.99). For the mildly frail and frail patients the overall pattern of results remained similar.

## Discussion

Our study shows that despite variations in rates of ICU admissions between northern and southern countries, no difference in in-hospital mortality was observed in frail COVID-19 patients.

Our results are in line with the scarce literature that indicates that the most severely frail patients from the northern regions are more often withheld from an ICU admission compared to the southern regions [12]. Given the neutral findings regarding in-hospital mortality, our data suggests that ICU admission might not or only to a limited extent contribute to survival rates in the frailest patients (CFS 6–9). Mildly frail patients (CFS 4–5) had a lower risk on in-hospital mortality in the southern countries compared to the northern countries, although no difference in ICU admission was observed. The mildly frail patients may represent a subgroup that has the most potential to improve outcomes, as they are likely to have sufficient physiological reserves to recover and return to their premorbid status when provided with appropriate critical care. In addition, it is possibly difficult for clinicians to classify patients as mildly frail while fit and severely frail are less disputable. It might be hypothesized that cultural differences between north and south European countries affect classification of mildly frail patients, especially since the CFS was assigned retrospectively. In southern countries, where living together with multiple generations is more common, physicians in the Emergency Department might classify these patients as mildly frail because they are community-dwelling and therefore presumed to be functional and relatively independent [13]. However, multigenerational living arrangements may mean that family members provide significant ADL and/or iADL support, potentially leading to an underestimation of frailty. Although we did not find evidence for this in the existing literature, this hypothesis warrants further investigation.

For the interpretation of our results, we must be careful since we were not able to incorporate a disease severity scale, such as APACHE SOFA, or SAPS, in order to assess differences in disease severity between regions. Since disease severity on the ICU is related with in-hospital mortality, this could help to explain our findings [14]. Nevertheless, the CFS is a patient-specific clinical judgement tool with an explicit role in triage during the pandemic. Furthermore, we were unable to collect data regarding ICU capacity among the participating centers, which possibly affected triage decisions. Moreover, the definition of an ICU bed is not standardized in Europe, and can even differ within countries. We tried to overcome this limitation by using a standardized definition from literature and excluding medium care beds from our analyses. Furthermore, the sensitivity analyses, in which the allocation of countries within regions was adjusted, showed no relevant differences in results, which strengthens our findings. Besides, in this study only patients admitted to the hospital in the first wave of the pandemic were included. More research is needed in additional waves of the pandemic to assess the effect of regional triage decisions on ICU admission and mortality.

We conclude that this brief report provides some support to current guidelines, to not only take into account age and comorbidity burden but also frailty status before potential ICU admission. As in-hospital mortality is comparable among fit and frail patients from northern and southern regions, upholding a higher threshold for ICU admission in particularly frail patients might help optimize prioritization of resources in a pandemic setting while also offering more options for palliative care instead of ICU admission.

## Electronic supplementary material

Below is the link to the electronic supplementary material.

Supplementary Material 1

## Data Availability

The individual, de-identified patient data that underlie the results reported in this short report (text and table) are available on reasonable request from the corresponding author (Julia Minnema) under certain conditions (with the consent of all participating centers and with a signed data access agreement).

## Abbreviations

ICU: Intensive Care Unit
CFS: Clinical Frailty Scale
SARS-CoV-2: Severe Acute Respiratory Syndrome COronaVirus 2
COMET: COvid MEdicaTion study
OR: Odds Ratio
CI: Confidence Interval
