# Correction: Regional differences in triage decisions affect hospital mortality among frail COVID-19 patients in the COvid MEdicaTion study

**DOI:** 10.1186/s12879-025-10702-2

**Published:** 2025-03-10

**Authors:** Julia Minnema, Roos Sablerolles, Janneke van Kempen, Hugo van der Kuy, Harmke Polinder-Bos, Bob van de Loo, Jorie Versmissen, Miriam L. Haaksma, Melvin Lafeber, Miriam C. Faes, the COMET research team^, Julia Minnema, Roos Sablerolles, Janneke van Kempen, Hugo van der Kuy, Harmke Polinder-Bos, Bob van de Loo, Jorie Versmissen, Melvin Lafeber, Miriam C. Faes, Ingrid van Haelst, Louise Andrews, Marleen Kemper, Roland van den Berg, Elise Slob, Firdaouss Boutkourt, Erik van Kan, Ronald Van Etten, Mariette Kappers, Peter van Wijngaarden, Anja Vos, Linda Hendriksen, Hugo de Wit, Loes Visser, Judith Derijks, Doris Haider, Nikolaus Lindner, Monika Schwap, Jos Tournoy, Lorenz Van der Linden, Morten Baltzer Houlind, Roberto Tessari, Paola Chessa, Marco Gambera, Isabella Martignoni, Laura Agnoletto, Margarida Falcao, Helena Farinha, Dina Mendes, Ines Carmo, Joana Soares, Fatima Falcao, Mariana Solano, Erica Viegas, Marta Miarons, Maria Queralt Gorgas, Cristina García Yubero, Kim Blum, Kim Keijzers, Silke Lim

**Affiliations:** 1 Department of Internal Medicine, Erasmus MC, University Medical Center Rotterdam, Rotterdam, the Netherlands; 2 Department of Hospital Pharmacy, Erasmus MC, University Medical Center Rotterdam, Rotterdam, the Netherlands; 3 Department of Geriatrics, Amphia Hospital, Breda, the Netherlands; 4 Digitalis Rx BV, Amsterdam, the Netherlands; 5 Department of Public Health and Primary Care, Leiden University Medical Centre, Leiden, The Netherlands; 6 University Network for the Care Sector South-Holland, Leiden University Medical Center, Leiden, The Netherlands

**Correction: BMC Infect Dis 25, 165 (2025)**

**https://doi.org/10.1186/s12879-025–10540-2**

Following publication of the original article [1], we were notified that the names of the collaborators in the COMET research team had not been correctly tagged in the article’s XML.

The original article has been corrected.
