# Supplementary material for: Regional differences in triage decisions affect hospital mortality among frail COVID-19 patients in the COvid MEdicaTion study
Source: BMC Infect Dis. 2025 Feb 4;25:165. doi: 10.1186/s12879-025-10540-2 (PMC11795989; doi:10.1186/s12879-025-10540-2)
Supplement: Supplementary file 1 — Supplementary Material 1 [file 12879_2025_10540_MOESM1_ESM.docx]

**SUPPLEMENTAL FILE**

**Supplemental List 1.**

List of authors COMET research team

**^*^**Consortium representative

Julia Minnema^1^, Roos Sablerolles^2^ , Janneke van Kempen^3^, Hugo van der Kuy^2*^, Harmke Polinder-Bos^1^, Bob van de Loo^4^, Jorie Versmissen^1,2^, Melvin Lafeber^1^, Miriam C. Faes^3^, Ingrid van Haelst^5^, Louise Andrews^6^, Marleen Kemper^7^, Roland van den Berg^7^, Elise Slob^7^, Firdaouss Boutkourt^8^, Erik van Kan^9^, Ronald Van Etten^3^, Mariette Kappers^3^, Peter van Wijngaarden^3^, Anja Vos^10^, Linda Hendriksen^11^, Hugo de Wit^12^, Loes Visser^13^, Judith Derijks^14^,Doris Haider^15^, Nikolaus Lindner^15^, Monika Schwap^15^, Jos Tournoy^16^, Lorenz Van der Linden^16^, Morten Baltzer Houlind^17^, Roberto Tessari^18^, Paola Chessa^19^, Marco Gambera^20^, Isabella Martignoni^20^, Laura Agnoletto^21^, Margarida Falcao^22^, Helena Farinha^22^, Dina Mendes^22^, Ines Carmo^22^, Joana Soares^23^, Fatima Falcao^23^, Mariana Solano^23^, Erica Viegas^23^, Marta Miarons^24^, Maria Queralt Gorgas^24^, Cristina García Yubero^25^, Kim Blum^26^, Kim Keijzers^27^, Silke Lim^27^

^1^ Department of Internal Medicine, Erasmus MC, University Medical Center Rotterdam, the Netherlands

^2^ Department of Hospital Pharmacy, Erasmus MC, University Medical Center Rotterdam, the Netherlands

^3^ Amphia Hospital, Breda, the Netherlands

^4^ Digitalis Rx BV, Amsterdam, the Netherlands

^5^ Noordwest Ziekenhuisgroep, Alkmaar, the Netherlands

^6^  Meander MC, Amersfoort, the Netherlands

^7^ Amsterdam UMC, AMC, Amsterdam, the Netherlands

^8^ Farmadam Apotheek, Amsterdam, the Netherlands

^9^ Gelre ziekenhuizen, Apeldoorn/Zutphen, the Netherlands

^10^ Treant Zorggroep, Emmen, the Netherlands

^11^ Tergooi hospital, Hilversum, the Netherlands

^12^ Canisius Wilhelmina Hospital, Nijmegen, the Netherlands

^13^ Haga Ziekenhuis, Den Haag, the Netherlands

^14^ Radboud UMC, Nijmegen, the Netherlands

^15^ Klinik Favoriten, Vienna, Austria

^16^ University Hospitals Leuven, Belgium

^17^ Copenhagen University Hospital Amager and Hvidovre, Denmark

^18^ IRCCS Ospedale Sacro Cuore Don Calabria, Negrar di Valpolicella, Italy

^19^ San Francesco Hospital Nuoro, Italy

^20^ Pederzoli Hospital, Peschiera del Garda, VR, Italy

^21^ Azienda Ulss 5 Veneto, Rovigo, Italy

^22^ Egas Moniz Hospital, Lisboa, Portugal

^23^ Hospital São Francisco Xavier, Lisboa, Portugal

^24^ Vall d’Hebron Hospital, Barcelona, Spain

^25^ University Hospital Infanta Sofía, San Sebastián de los Reyes, Spain

^26^ FHP Kantonsspital Aarau, Aarau, Switzerland

^27^ Cantonal Hospital of Lucerne, Lucerne, Switzerland

**Supplemental Table 1.** Logistic regression analysis for ICU admission (sensitivity analysis)

|  | **North** | **South** |
| --- | --- | --- |
| **Fit (CFS 1 – 3)** |  |  |
| Model 1: crude analysis | Ref. | 0.41 (0.32 – 0.53) |
| Model 2: model 1 + adjusted for age and sex | Ref. | 0.44 (0.34 – 0.56) |
| **Mildly frail (CFS 4 – 5)** |  |  |
| Model 1: crude analysis | Ref. | 0.93 (0.60 – 1.43) |
| Model 2: model 1 + adjusted for age and sex | Ref. | 0.86 (0.55 – 1.36) |
| **Frail (CFS 6 – 9)** |  |  |
| Model 1: crude analysis | Ref. | 1.51 (1.03 – 2.23) |
| Model 2: model 1 + adjusted for age and sex | Ref. | 1.69 (1.13 – 2.54) |

Estimates are presented as Odds ratios (95% confidence interval)

North: Belgium, Denmark, Great Britain, the Netherlands;

South: Austria, Switzerland, Germany, France, Italy, Portugal, Spain

**Supplemental Table 2.** Logistic regression analysis for in-hospital mortality (sensitivity analysis)

|  | **North** | **South** |
| --- | --- | --- |
| **Fit (CFS 1 – 3)** |  |  |
| Model 1: crude analysis | Ref. | 0.60 (0.42 – 0.86) |
| Model 2: model 1 + adjusted for age and sex | Ref. | 0.68 (0.47 – 0.99) |
| **Mildly frail (CFS 4 – 5)** |  |  |
| Model 1: crude analysis | Ref. | 0.25 (0.16 – 0.37) |
| Model 2: model 1 + adjusted for age and sex | Ref. | 0.28 (0.18 – 0.44) |
| **Frail (CFS 6 – 9)** |  |  |
| Model 1: crude analysis | Ref. | 1.31 (0.90 – 1.90) |
| Model 2: model 1 + adjusted for age and sex | Ref. | 1.14 (0.76 – 1.70) |

Estimates are presented as Odds ratios (95% confidence interval)

North: Belgium, Denmark, Great Britain, the Netherlands;

South: Austria, Switzerland, Germany, France, Italy, Portugal, Spain
